# Impact of the Second Epidemic Wave of SARS-CoV-2: Increased Exposure of Young People

**DOI:** 10.3389/fpubh.2021.715192

**Published:** 2021-07-26

**Authors:** Lisandru Capai, Shirley Masse, Toscane Fourié, Dorine Decarreaux, Jean Canarelli, Marie-Helene Simeoni, Abdennour Amroun, Souand Mohammed-Ali, Paola Mariela Saba Villarroel, Xavier de Lamballerie, Rémi Charrel, Alessandra Falchi

**Affiliations:** ^1^UR 7310, Laboratoire de Virologie, Université de Corse, Corte, France; ^2^Unité des Virus Émergents: Aix Marseille University, IRD 190, INSERM 1207, IHU Méditerranée Infection, Marseille, France; ^3^Laboratoire de biologie médicale, CCF, Ajaccio, France; ^4^Laboratoire de Biologie médicale 2A2B, Corte, France

**Keywords:** seroprevalence, SARS-CoV-2, antibodies, France, enzyme-linked immunosorbent assay, seroneutralization

## Abstract

We aimed to use serological surveillance based on serial cross-sectional sampling of residual sera obtained from clinical laboratories to compare the differences in age and sex profiles of infected persons in the first and second waves of SARS-CoV-2 in Corsica, France. Residual sera were obtained, including samples from individuals of all ages collected for routine screening or clinical management by clinical laboratories. All the sera collected were tested for the presence of anti-SARS-CoV-2 IgG using a kit for semi-quantitative detection of IgG antibodies against the S1 domain of the viral spike protein (ELISA-S). Samples that were borderline and positive in ELISA-S were tested with an in-house virus neutralization test. During the second-wave period, we collected between 6 November, 2020 and 12 February, 2021, 4,505 sera from patients aged 0–101 years (60.4% women). The overall weighted seroprevalence of residual sera collected during the second-wave period [8.04% (7.87–9.61)] was significantly higher than the overall weighted seroprevalence estimated at the end of the first wave between 16 April and 15 June, 2020 [5.46% (4.37–7.00)] (*p*-value = 0.00025). Ninety-eight (30.1%) of the 326 samples tested in the VNT assay had a positive neutralization antibody titer. Estimated seroprevalence increased significantly for men [odds ratio (OR) OR = 1.80 (1.30–2.54); *p*-value = 0.00026] and for people under 30 years of age [OR = 2.17 (1.46–3.28); *p*-value = 0.000032]. This increase was observed in young adults aged 20–29 years among whom antibody frequencies were around four-fold higher than those observed at the end of the first wave. In conclusion, our seroprevalence estimates, including the proportion of the participants who had produced neutralizing antibodies, indicate that in February, 2021 the population of Corsica was still far from being protected against SARS-Cov-2 by “herd immunity.”

## Introduction

In France, during the first wave of severe acute respiratory syndrome coronavirus 2 (SARS-CoV-2) infections (18th March to 16th June 2020), the government decreed a national lockdown from 17 March to 11 May, 2020, to mitigate dissemination. Studies have highlighted differences between regions in the seroprevalence of anti-SARS-CoV-2 IgG, ranging from 1 to 3% in the least affected area (Nouvelle Aquitaine) to 9–11% in Ile-de-France (1). The nationwide seroprevalence of SARS-CoV-2 antibodies was estimated at 0.41% mid-March, 4.14% mid-April and 4.93% mid-May (2).

Our previous Corsica-based seroprevalence study estimated that in mid-June 5.46% of the Corsican population had been infected by SARS-CoV-2 (3). Consistent with previous population-based serosurveys (4–6), we reported noticeable age-related differences, with a lower seroprevalence in children <9 year-old compared with adolescents and adults. This low seroprevalence has been explained as a lower susceptibility of young children to SARS-CoV-2 infection than with adults. Adolescents appear to have similar susceptibility to adults (6), but a lower seroprevalence because of the strict lockdown that limited the exposure of school-age populations, whereas adult-aged essential professionals have continued to be exposed in the community (7).

In France, the number of patients increased again after a decrease during summer, suggesting a second wave of COVID-19 outbreak (2020w35–2020w52). The second wave was managed differently from the first one, with a “light lockdown,” expanded testing resources and the implementation of health protocols in schools that, unlike the closure during the first wave, were kept open.

Thus, to evaluate whether age disparities in those infected could be observed over time during the second wave, we implemented a serological surveillance based on serial cross-sectional sampling using sera obtained from clinical laboratories across Corsica. We estimated by age and sex the fraction of the Corsican population infected with SARS-CoV-2 over time as well as the proportion of individuals having developed neutralizing antibodies.

## Materials and Methods

### Sample Design

French regulations stipulate that serum samples collected for diagnostic purpose after medical prescription must be stored for 1 year. Afterwards, they can be discarded or used for research purposes under the specific terms of the law (Reference Method MR 004). such samples will be denominated as “residual sera” (RS) in the current study. RS consisted of samples from individuals of all ages collected for routine screening or clinical management by 13 clinical laboratories located in five areas between 6 November, 2020 and 12 February, 2021 (Supplementary Figure 1). In the first 2 weeks of each month of collection, we selected a convenient sample of RS from the whole set to achieve representative sample numbers for each of the 10-year age groups (0–9 years to ≥90 years) on the basis of the real Corsican distribution (8). For each RS we collected age, sex and geographical localization of the patient.

### Laboratory Analysis

RS were tested for the presence of anti-SARS-CoV-2 IgG using the EUROIMMUN enzyme immunoassay kit for semi-quantitative detection of IgG antibodies against the S1 domain of the viral spike protein (ELISA-S) (reference: EI 2606-9601 G; EUROIMMUN, Bussy-Saint Martin, France). According to the manufacturer's instruction, a result was considered borderline if the ratio was between ≥0.8 and <1.1 and positive if the sample ratio was ≥1.1. In all the positive and borderline samples, neutralizing antibodies were analyzed using a Virus Neutralization Test (VNT) as previously described (9). VeroE6 cells cultured in 96-well microplates, 100 Fifty-percent tissue culture infective dose (TCID50) of the SARS-CoV-2 strain BavPat1 (courtesy of Pr. Drosten, Berlin), and serial dilutions of serum (1/20–1/160) were used. Dilutions associated with cytopathic effect (CPE) were considered negative (no neutralization) and those with no CPE at day 4 post-infection were considered positive (complete neutralization). The neutralization titer refers to the highest dilution of serum with a positive result. Specimens with a VNT titer 40 were considered positive.

### Statistical Analysis

According to overall weighted seroprevalence observed in RS at mid-June (3) and at the end of October (clinical laboratory data) a minimum sample size of 1,109 was calculated assuming an *a priori* 7% anti-SARS-CoV-2 IgG seroprevalence (data estimated in November, 2020), a confidence in the estimate of 95%, a maximum allowable error in the prevalence of 1.5% and a Corsican population size of 344,679 habitants based on the latest French census data (8, 10).

**Figure 1:**
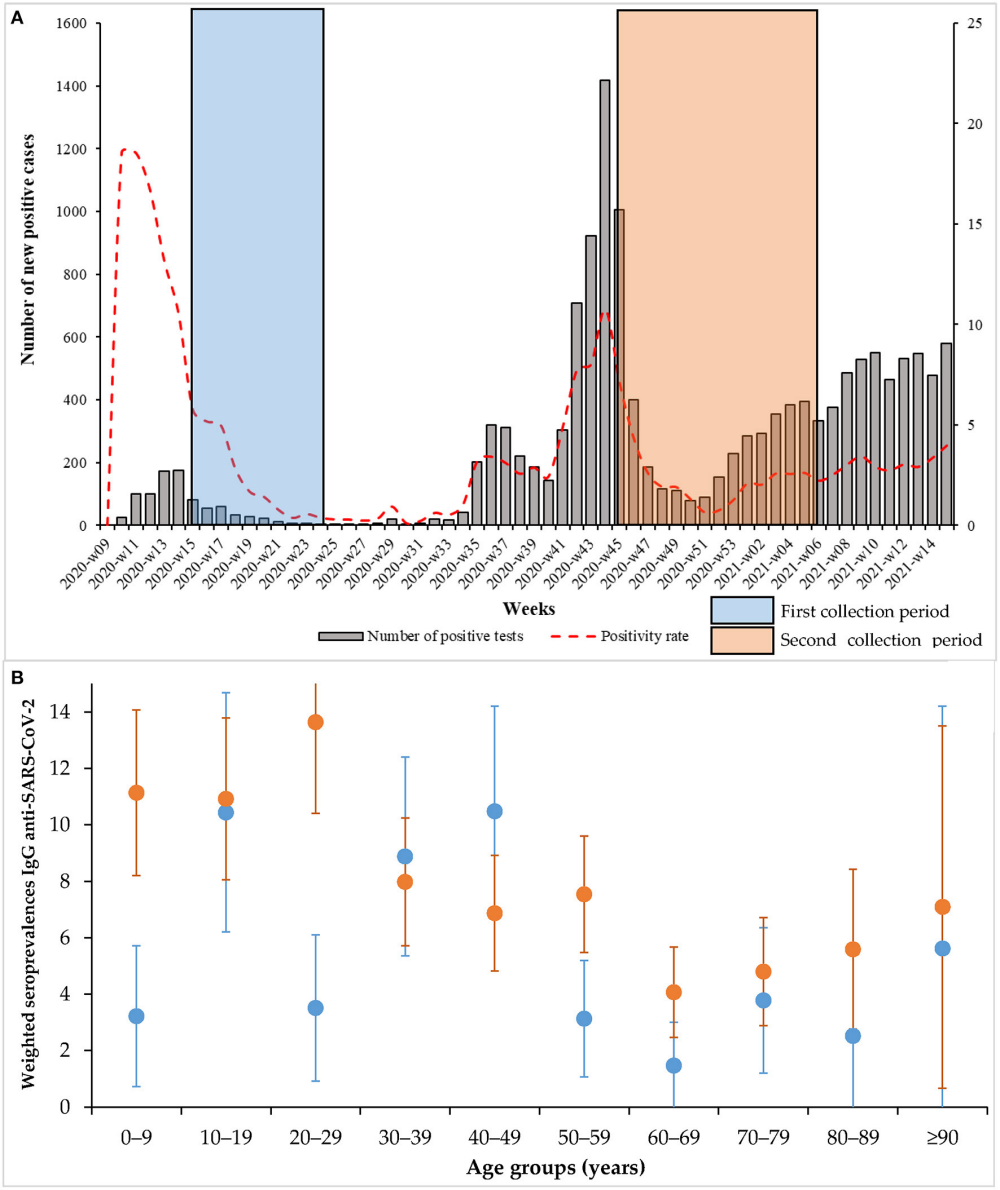
**(A)** Positivity rate and number of positive cases by week; **(B)** Weighted seroprevalences according to the different age groups (blue: first collection period, orange: second period. The bars correspond to the 95% confidence intervals).

Descriptive statistical methods were used for age and sex. Age was described as the median with interquartile ranges (IQRs). Categorical data were reported as percentages. Anti-SARS-CoV-2 IgG seroprevalence and its 95% exact binomial confidence intervals (CIs) were estimated. Seroprevalence by age group, sex and geographical areas was weighted according to the proportions observed in the general population obtained from the French national institute of statistics and economic studies (Supplementary Figure 2) (8). We also calculated an “adjusted seroprevalence” taking into account the specificity and the sensibility of the Euroimmun assay (99.8 and 90.3%, respectively according to the manufacturer's data), as described in a recent seroprevalence study in Switzerland (4). Associations of the presence of anti-SARS-CoV-2 IgG with sex and age and location were tested using the χ^2^-test or Fisher's exact-test. The odds ratio (OR) was used to describe the risk of sera being positive in ELISA-S compared with that of non-positive ELISA-S results. Statistical significance was set at a *p*-value < 0.05. Weighted seroprevalences of residual sera collected during the second-wave period were compared with the weighted seroprevalences estimated during the first wave between 16 April and 15 June, 2020 (Figure 1A) (3). All statistical analyses were performed using R software version 3.6.1 (R Foundation, Vienna, Austria).

### Ethics Statement, Collection Data, and Statistical Analysis

No nominative or sensitive data on participants were collected. This seroprevalence study falls within the scope of the French Reference Methodology MR-004 according to 2016–41 law dated 26 January, 2016 on the modernization of the French health system.

## Results

Between 6 November, 2020 and 12 February, 2021, we collected 4,505 RS from patients aged 0–101 years (60.4% women) (Supplementary Table 1) of whom 326 (7.2%) were positive for anti-SARS-CoV-2 IgG. Month-weighted seroprevalences increased in a non-significant way from 6.75 (5.15–8.34) to 8.68% (6.92–10.00) between 6 November and 12 February, respectively with an overall seroprevalence of 8.04% (7.87–9.61) (Table 1). The adjusted seroprevalences according to the sensibility and the specificity of the seroassay were also presented in the Table 1. The overall adjusted seroprevalence during the first period was 5.84 and 8.70% during the second period.

**Table 1:** Overall seroprevalence during the first and second national epidemic waves and univariate analysis of variables.

		**First epidemic wave**			**Second epidemic wave (November to February)**			**Univariate analysis of variables during the second wave**		**Comparative analysis between first and second waves**	
		**Overall *N***	**Weighted Seroprevalence (%) (95% CI)**	**Adjusted seroprevalence**	**Overall *N***	**Weighted Seroprevalence (%) (95% CI)**	**Adjusted seroprevalence**	**OR (95% CI)**	***p*-value**	**OR (95% CI)**	***p*-value**
Overall		1,973	5.46 (4.37–7.00)	5.84	4,407.70	8.04 (7.24–8.84)	8.70			**1.51 (1.20–1.90)**	0.00025^*^
Sex	Women	1,017.35	5.74 (4.31–7.17)	6.15	2,300.65	7.06 (6.01–8.11)	7.61	1.29 (0.94–1.61)	0.12	1.25 (0.91–1.74)	0.17
	Men	955.48	5.15 (3.75–6.56)	5.49	2,161.59	8.88 (7.68–10.08)	9.63			**1.80 (1.30–2.54)**	0.00026^*^
Age groups (years)	0–9	193.05	3.22 (0.73–5.71)	3.35	441.14	11.14 (8.20–14.07)	12.14	1.46 (0.54–3.95)	0.46	**3.89 (1.63–11.31)**	0.00062^*^
	10–19	199.79	10.44 (6.20–14.68)	11.37	455.26	10.92 (8.06–13.79)	11.90	1.43 (0.83–2.46)	0.2	1.05 (0.60–1.90)	1
	20–29	191.59	3.51 (0.90–6.10)	3.67	434.98	13.64 (10.42–16.87)	14.92	**1.81 (1.13–2.91)**	0.015^*^	**4.14 (1.84–10.96)**	0.0001^*^
	30–39	249.66	8.88 (5.35–12.40)	9.63	554.31	7.98 (5.72–10.24)	8.63	Reference		0.89 (0.51–1.61)	0.68
	40–49	258.47	10.48 (6.74–14.21)	11.41	584.88	6.87 (4.82-8.92)	7.40	0.85 (0.53–1.35)	0.48	0.63 (0.37–1.09)	0.096
	50–59	274.3	3.13 (1.07–5.19)	3.25	622.94	7.54 (5.47–9.61)	8.15	0.95 (0.61–1.46)	0.81	**2.41 (1.15–5.68)**	0.016^*^
	60–69	257.6	1.47 (0.00–2.94)	1.41	581.13	4.07 (2.46–5.67)	4.30	**0.49 (0.29–0.83)**	0.00085^*^	2.73 (0.92–10.93)	0.061
	70–79	209.46	3.78 (1.20–6.36)	3.97	474.2	4.8 (2.87–6.72)	5.11	**0.59 (0.36–0.95)**	0.031^*^	1.29 (0.54–3.39)	0.69
	80–89	111.32	2.52 (0.00–5.42)	2.57	252.14	5.59 (2.75–8.42)	5.98	0.69 (0.42–1.13)	0.14	2.13 (0.58–11.81)	0.29
	≥90	27.59	5.62 (0.00–14.21)	6.02	61.27	7.09 (0.66–13.51)	7.65	0.89 (0.48–1.63)	0.7	0.91 (0.12–10.70)	1
	<30	584.43	5.78 (3.89–7.67)	6.19	1,331.39	11.82 (10.09–13.55)	12.90	**2.015 (1.38–2.94)**	0.000282^*^	**2.17 (1.46–3.28)**	0.000032^*^
	≥30	1,388.57	5.32 (4.14–6.50)	5.68	3,130.77	6.24 (5.39–7.09)	6.70			0.85 (0.63–1.12)	0.25
Geographical areas	Grand Ajaccio	1,442.65	6.29 (5.04–7.55)	6.76	1,942.57	7.49 (6.32–8.66)	8.09	Reference		1.21 (0.91–1.60)	0.19
	Plaine Orientale				463.32	8.71 (6.14–11.28)	9.45	1.18 (0.58–2.41)	0.7		
	Cortenais	530.18	3.18 (1.69–4.68)	3.31	1,249.67	7.97 (6.47–9.47)	8.62	1.07 (0.79–1.46)	0.71	**2.62 (1.54-4.73)**	0.000097^*^
	Balagne				727.78	8.54 (6.51–10.57)	9.26	1.15 (0.80–1.67)	0.52		
	Grand Sud				78.83	6.4 (1.00–11.81)	6.88	0.85 (0.37–1.92)	0.74		

*OR, Odd ratios; CI, Confidential Interval*.

*Bold values are significant values statistically (Odds ratio and p-values)*.

* *means significant values*.

We observed a similar overall seroprevalence in both sexes, 7.06% (6.01–8.11) for women and 8.88 (7.68–10.08) for men (*p* = 0.12) (Table 1). Persons under 30 years of age had a higher seroprevalence [11.82% (10.09–13.55)] than people older than 30 years [6.24% (5.39–07.09)] [OR = 2.02 (1.38–2.94); *p*-value = 0.000282] (Table 1). Seroprevalence values did not differ significantly between the five areas (Table 1).

The overall weighted seroprevalence estimated during the second-wave period of collection [8.04% (7.87–9.61)] was significantly higher than the overall weighted seroprevalence estimated at the end of the first wave collection period [5.46% (4.37–7.00)] [OR = 1.51 (1.20–1.90); *p*-value = 0.00025] (Table 1). Seroprevalence estimates increased significantly during the second wave compared with the first wave for men [OR = 1.80 (1.30–2.54); *p*-value = 0.00026] and for people under 30 years of age [OR = 2.17 (1.46–3.28); *p*-value = 0.000032]. This increase was observed in children aged <9 years and in young adults aged 20–29 years (Table 1 and Figure 1B). As shown in Table 1 and Figure 1B, the estimated seroprevalence in all age groups was higher during the second period of collection than during the first (Table 1 and Figure 1), except for people aged 30–49 years. However, the increase was significant in only three age groups: 0–9, 20–29, and 50–59 years.

The area of central Corsica showed the greatest increase [7.97% (6.47–9.47)] compared with the seroprevalence estimated at the end of the first wave [3.18% (1.69–4.68)] [OR = 2.62 (1.54–4.73); *p*-value = 0.000097]. The different results obtained by month during the study are presented in the Supplementary Table 2 and Supplementary Figure 3.

During the second-wave collection period, 98 (30.1%) of the 326 samples tested in the VNT assay had a positive neutralization antibody titer (VNT titer ≥ 40) (Supplementary Table 3). VNT titres did not differ significantly between age groups or sexes. The overall prevalence of samples above the cut-off (titer 40) (98/4,505) was 2.17% (1.75–2.60).

## Discussion

We observed a significant increase in overall seroprevalence of anti-SARS-CoV-2 IgG in Corsica during the collection period of November, 2020 to February, 2021 compared with results obtained at the end of the first wave (3), 8.04 (CI 95 7.24–8.84) vs. 5.46 (CI 95 4.37–7.00). This increase was mainly observed in in young adults aged 20–29 years: in these age classes, seroprevalence was four-fold higher than at the end of the first wave. This result is not surprising because the increase in community transmission provided more opportunities for the introduction of the virus (e.g., educational settings workplaces, sports activities). Schools and universities, closed during the first wave, reopened at the beginning of September, and remained open until 18 December, with the exception of a 2-week autumn break. Because the participants underwent serology testing between November 6, 2020 and February 12, 2021, the results reported here mainly reflect the circulation of SARS-COV-2 since summer 2020 because IgG responses to the spike protein are stable for at least 6 months (11).

Following the first epidemic wave, similar IgG anti-SARS-CoV-2 seroprevalences were observed (about 5%) in different European countries (Spain, Italy, Scotland, Switzerland, continental France) (2, 12–16). A lower seroprevalence value was estimated in Germany during the same period, 0.91% (17). No european data are available for the second wave in the non-hospitalized population.

The present study did not detect any significant sex-related difference in SARS-CoV-2 seroprevalence during the second wave. However, males showed a significant 80% increase in weighted seroprevalence between the first and second wave. According to the European Center for Disease Prevention and Control, critical illness due to COVID-19 is seen 2.7 times more frequently in men than in women (18). This sex-related difference in seroprevalence might be caused by unknown factors underlying patterns of transmission or to different behaviors, but might also have a biological origin if differences in immunological response or severity of disease exist between sexes. However, other studies have reported a higher seroprevalence in women than in men, so the issue remains controversial (19, 20).

Consistent with other studies, the lower rates were observed in age classes above 60 years (4, 16, 21). Since people in these age classes are the main risk group for COVID-19 severe forms, the observed low prevalence may result from their tendency to minimize social interactions. Another reason is the reduced efficacy of the immune response that could account for antibody fading (22, 23). Of course, it is possible that the two mechanisms are combined.

Different points are important to discuss regarding our results. First, because seroconversion after SARS-CoV-2 infection takes several days (24), recent infections could be missed at the individual level. However, at the population level, this lag between IgG detection and the day of infection is taken into account on the results of the period we presented. Indeed, our collection periods were carried out after the epidemic waves. Second, children and adolescents are less likely to undergo routine blood testing, therefore their representation in the study was relatively low compared with their proportion in the general Corsican population. It is therefore important to note that we have weighted our data and that few studies have been able to measure seroprevalence in children and adolescents. Third, seroprevalence is a dynamic parameter because some participants lose antibodies and may appear seronegative despite having had the virus. Our results may therefore underestimate the true impact of the infections. Moreover, generally, seroreversion reflects the part of individuals that fall below the sensitivity threshold of the serological kits used and does not reflect the true part of people that completely have lost their antibodies (25). Serological status is only a partial indication of immunity to SARS-CoV-2 infection because other non-specific or T-cell-mediated cellular responses may exist to confer long-term immunity (9). Finally, the current survey population was composed of people in which blood test was prescribed for medical reasons (e.g., chronic diseases), they could have been more careful and decreased their exposure to the virus, which resulted in an underestimation of seroprevalence so they may not reliably represent the whole Corsican population. However, there is no evidence to suggest a greater susceptibility to COVID-19 in people undergoing blood tests in clinical laboratories and several nationwide studies have employed this biological sample (“residual sera”) to describe SARS-CoV-2 circulation (2, 26, 27).

In conclusion, our seroprevalence estimates, including the proportion of the participants who had produced neutralizing antibodies, indicate that in February, 2021 the population of Corsica was still far from being protected against SARS-Cov-2 by “herd immunity.” In the present study, one-third of the participants had produced SARS-CoV-2 neutralizing antibodies with a titer ≥ 40. At the end of January, 2021, a broad vaccination program was launched in Corsica, aimed at vaccinating the entire population aged over 12 years against SARS-CoV-2 before the end of the summer 2021. There is growing evidence that vaccines could reduce the transmission of SARS-CoV-2, so vaccination of children could also have a beneficial effect on the wider community. The emergence of more rapidly spreading variants, as well as increased adult vaccination rates, means that children and adolescents may soon contribute more to the spread. However, the unequal distribution of vaccines worldwide, particularly in non-industrialized countries, also raises other questions at the ethical and virological level. Indeed, is it ethical to vaccinate a population that is less at risk to severe COVID-19, while adults at risk are still unvaccinated in other countries? This problem may also have repercussions concerning the rapid appearance of new variants in countries with very low vaccination coverage and which could subsequently escape vaccine protection.

## Data Availability Statement

The raw data supporting the conclusions of this article will be made available by the authors, without undue reservation.

## Author Contributions

LC, AF, RC, M-HS, and JC conceptualized the study. LC wrote the original draft. JC and M-HS were the investigators of the study and managed the laboratories for the collect of samples. LC, DD, and SM collected blood samples, managed the ELISA tests, and interpreted the results. TF, AA, SM-A, and PS managed the Virus neutralization tests and interpreted the results. LC and AF realized the statistical analysis. AF, RC, and XL supervised the study and revised the manuscript. All authors contributed to the article and approved the submitted version.

## Conflict of Interest

The authors declare that the research was conducted in the absence of any commercial or financial relationships that could be construed as a potential conflict of interest.

## Publisher's Note

All claims expressed in this article are solely those of the authors and do not necessarily represent those of their affiliated organizations, or those of the publisher, the editors and the reviewers. Any product that may be evaluated in this article, or claim that may be made by its manufacturer, is not guaranteed or endorsed by the publisher.
