# Supplementary material for: Impact of the Second Epidemic Wave of SARS-CoV-2: Increased Exposure of Young People
Source: Front Public Health. 2021 Jul 26;9:715192. doi: 10.3389/fpubh.2021.715192 (PMC8350127; doi:10.3389/fpubh.2021.715192)
Supplement: Supplementary file 1 [file Table_1.DOCX]

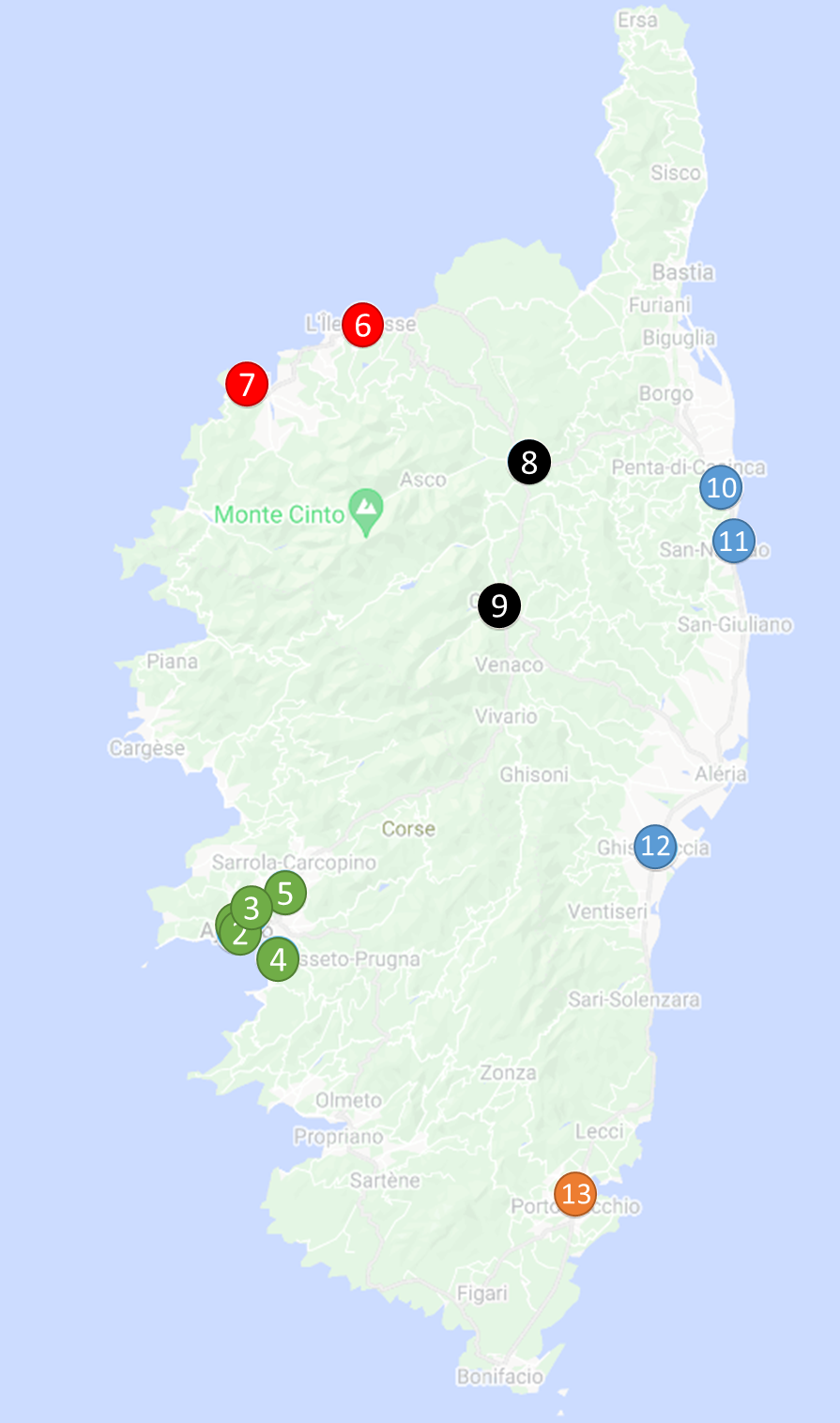
Supplementary material and data

**Supplementary Figure 1:** Distribution of the 13 participating laboratories (1–5: Grand Ajaccio with green circles; 6, 7: Balagne with red circles; 8, 9: Center of Corsica with black circles; 10–12: Plaine Orientale with blue circles and 13: Grand Sud with orange circle).

**Supplementary Figure 2:** Age and sex distribution of the Corsican population (**A**) and the studied population (**B**).

**Supplementary Table 1:** Description of the sampled population according to age, sex and geographical areas of Corsica

|  | | Total |
| --- | --- | --- |
| Sex | Women | 2,723 |
|  | Men | 1,782 |
| Age groups | 0–9 | 58 |
|  | 10–19 | 251 |
|  | 20–29 | 404 |
|  | 30–39 | 540 |
|  | 40–49 | 596 |
|  | 50–59 | 695 |
|  | 60–69 | 582 |
|  | 70–79 | 638 |
|  | 80–89 | 519 |
|  | > 90 | 222 |
| Geographical Areas | Grand Ajaccio | 1,867 |
|  | Cortenais | 1,339 |
|  | Balagne | 718 |
|  | Grand Sud | 90 |
|  | Plaine Orientale | 491 |
| Overall | | 4,505 |

**Supplementary Table 2:** Weighted seroprevalences according to the month of collection, sex and age group

|  | | **November 2020** | | | | **December 2020** | | | | **January 2021** | | | | **February 2021** | | | |
| --- | --- | --- | --- | --- | --- | --- | --- | --- | --- | --- | --- | --- | --- | --- | --- | --- | --- |
|  |  | **Overall** | **Weighted seroprevalences** | | | **Overall** | **Weighted seroprevalences** | | | **Overall** | **Weighted seroprevalences** | | | **Overall** | **Weighted seroprevalences** | | |
|  |  |  | **Number of positives** | **Seroprevalence (%)** | **[95% CI]** |  | **Number of positives** | **Seroprevalence (%)** | **[95% CI]** |  | **Number of positives** | **Seroprevalence (%)** | **[95% CI]** |  | **Number of positives** | **Seroprevalence (%)** | **[95% CI]** |
|  | Overall | 946.79 | 63.88 | 6.75 | [5.15–8.34] | 1,006.90 | 87.36 | 8.68 | [6.94–10.41] | 1,199.93 | 97.11 | 8.09 | [6.55–9.64] | 1,254.08 | 106.05 | 8.46 | [6.92–10.00] |
| **Sex** | Women | 488.39 | 28.86 | 5.91 | [3.82–8.00] | 519.08 | 41.96 | 8.08 | [5.74–10.43] | 646.47 | 54.25 | 8.39 | [6.25–10.53] | 646.71 | 37.34 | 5.77 | [3.98–7.57] |
|  | Men | 458.40 | 35.02 | 7.64 | [5.21–10.07] | 487.82 | 45.40 | 9.31 | [6.73–11.88] | 608.00 | 42.86 | 7.05 | [5.01–9.08] | 607.37 | 68.71 | 11.31 | [8.79–13.83] |
| **Age groups (years)** | 0–9 | 92.72 | 17.05 | 18.39 | [10.50–26.27] | 102.89 | 8.30 | 8.06 | [2.80–13.33] | 122.76 | 7.93 | 6.46 | [2.11–10.81] | 122.76 | 15.86 | 12.92 | [6.99–-18.85] |
|  | 10–19 | 95.84 | 11.72 | 12.23 | [5.67–18.79] | 106.12 | 15.02 | 14.15 | [7.52–20.79] | 126.65 | 11.98 | 9.46 | [2.89–12.05] | 126.65 | 11.00 | 8.68 | [3.78–13.59] |
|  | 20–29 | 91.48 | 7.76 | 8.48 | [2.77–14.19] | 100.76 | 17.72 | 17.59 | [10.15–25.02] | 121.38 | 17.83 | 14.69 | [8.39-20.99] | 121.36 | 16.03 | 13.21 | [7.18–19.23] |
|  | 30–39 | 119.91 | 3.37 | 2.81 | [0.00–5.77] | 116.94 | 8.48 | 7.25 | [2.55–11.95] | 158.69 | 15.86 | 9.99 | [5.33–14.66] | 158.77 | 16.53 | 10.41 | [5.66–15.16] |
|  | 40–49 | 123.99 | 6.42 | 5.18 | [1.28–9.08] | 132.03 | 8.74 | 6.62 | [2.38–10.86] | 164.40 | 8.97 | 5.46 | [1.98–8.93] | 164.46 | 16.07 | 9.77 | [5.23–14.31] |
|  | 50–59 | 132.11 | 3.74 | 2.83 | [0.00–5.66] | 140.81 | 8.69 | 6.17 | [2.20–10.15] | 175.40 | 19.79 | 11.28 | [6.60–15.97] | 174.62 | 14.74 | 8.44 | [4.32–12.56] |
|  | 60–69 | 123.83 | 6.16 | 4.97 | [1.15–8.80] | 129.15 | 7.24 | 5.61 | [1.64–9.57] | 164.10 | 5.85 | 3.57 | [0.73–6.40] | 164.05 | 4.38 | 2.67 | [0.20–5.14] |
|  | 70–79 | 100.06 | 4.24 | 4.24 | [0.29–8.18] | 107.26 | 6.96 | 6.49 | [1.83–11.15] | 133.44 | 5.20 | 3.90 | [0.61–7.18] | 133.44 | 6.35 | 4.76 | [1.15–8.37] |
|  | 80–89 | 53.52 | 3.42 | 6.39 | [0.00-12.94] | 57.46 | 4.61 | 8.01 | [1.00–15.05] | 70.55 | 1.85 | 2.63 | [0.00–6.35] | 70.62 | 4.21 | 5.96 | [0.44–11.48] |
|  | ≥90 | 13.33 | 0.00 | 0.00 | / | 13.48 | 1.61 | 11.98 | [0.00–29.26] | 17.10 | 1.84 | 10.77 | [0.00–25.45] | 17.37 | 0.89 | 5.12 | [0.00–15.49] |
|  | <30 | 280.04 | 36.53 | 13.04 | [9.10–16.99] | 309.78 | 41.04 | 13.25 | [9.47–17.02] | 370.79 | 37.74 | 10.18 | [7.10–13.26] | 370.77 | 42.89 | 11.57 | [8.31–14.82] |
|  | ≥30 | 666.75 | 27.35 | 4.10 | [2.60–5.61] | 697.12 | 46.33 | 6.65 | [4.80–8.49] | 883.68 | 59.37 | 6.72 | [5.07–8.37] | 883.31 | 63.16 | 7.15 | [5.45–8.85] |

**Supplementary Figure 3:** Seroprevalences of anti-SARS-CoV-2 IgG by age group (weighted population) during the four months of collection (November to February).

**Supplementary Table 3:** Results of the virus neutralization test (VNT)

|  | **First wave** | | **November** | | **December** | | **January** | | **February** | | **Second wave** | | **VNT Interpretation** |
| --- | --- | --- | --- | --- | --- | --- | --- | --- | --- | --- | --- | --- | --- |
| **VNT Titre** | **Number of samples** | **%** | **Number of samples** | **%** | **Number of samples** | **%** | **Number of samples** | **%** | **Number of samples** | **%** | **Number of samples** | **%** |  |
| **Negative** | **67** | 47.9 | **26** | 55.32 | **58** | 68.24 | **60** | 60.61 | **50** | 54.35 | **194** | 60.06 | n (%)  Below the cut-off 228 (70.6%) |
| **20** | **14** | 10.0 | **2** | 4.26 | **4** | 4.71 | **12** | 12.12 | **16** | 17.39 | **34** | 10.53 |  |
| **40** | **24** | 17.1 | **5** | 10.64 | **5** | 5.88 | **10** | 10.10 | **8** | 8.70 | **28** | 8.67 | Above the cut-off  95 (29.4%) |
| **80** | **9** | 6.4 | **4** | 8.51 | **8** | 9.41 | **6** | 6.06 | **9** | 9.78 | **27** | 8.36 |  |
| **160** | **26** | 18.6 | **10** | 21.28 | **10** | 11.76 | **11** | 11.11 | **9** | 9.78 | **40** | 12.38 |  |
| **Total** | **140** | 100.0 | **47** | 100.00 | **85** | 100.00 | **99** | 100.00 | **92** | 100.00 | **323** | 100.00 |  |
